# Quality of Outcome Reporting for Older Subgroups in FDA Registration Gastroesophageal Cancer Trials (2010–2024): A Systematic Review

**DOI:** 10.3390/cancers18172747

**Published:** 2026-08-24

**Authors:** Ilse Trip, Martha Kormi, Elizabeth Smyth, Russell D. Petty, Mark A. Baxter

**Affiliations:** 1 Tayside Cancer Centre, Ninewells Hospital and Medical School, NHS Tayside, Dundee DD1 9SY, UK; marthawatson@doctors.org.uk (M.K.); r.petty@dundee.ac.uk (R.D.P.); m.z.baxter@dundee.ac.uk (M.A.B.); 2 Department of Oncology, Oxford University Hospitals NHS Foundation Trust, Oxford OX3 7LE, UK; elizabeth.smyth2@nhs.net; 3 Division of Cancer Research, Ninewells Hospital and Medical School, University of Dundee, Dundee DD1 9SY, UK

**Keywords:** gastroesophageal cancer, geriatric oncology, clinical trials, outcome measures

## Abstract

**Background**: Gastroesophageal adenocarcinoma is predominantly a disease of older adults. However, older adults are underrepresented in randomised controlled trials (RCTs), and outcomes specific to this subgroup are underreported. Clinicians therefore extrapolate data from younger, fitter trial populations, which can lead to over- and undertreatment of older cancer patients. This study examines the inclusion and completeness of outcome reporting for older patients in practice-changing trials in gastroesophageal cancer. **Methods**: RCTs were identified from United States Food and Drug Administration (accelerated) approvals between 2010 and 2024. The primary study report, clinicaltrial.gov repository and all relevant secondary publications were assessed for the level of reporting of outcomes. Efficacy outcomes were reported as complete, partial, qualitative or quantitative based on availability of sample size, effect size and measures of precision. Baseline characteristics, toxicity outcomes and health-related quality of life (HRQOL) outcomes were assessed using minimum data thresholds. **Results**: In total, 13 trials, 53 publications and 13 repository webpages were assessed. A total of 9320 patients were included, of which 40.1% were classified as older adults. Among the patients, 42.2% had an Eastern Cooperative Oncology Group (ECOG) Performance Status (PS) of 0, with all but one trial excluding patients with an ECOG PS of 2 (*n* = 63). Baseline characteristics and toxicity outcomes for older adults were fully reported in only two trials (15.4%). Reporting of HRQOL outcomes (25.0%), secondary (20.0%) and primary endpoints (66.7%) was more complete. Overall survival (76.9%) and progression-free survival (67.0%) were the most commonly reported primary or secondary endpoints. **Conclusions**: This study highlights deficits in the outcome reporting of older cancer patients in GOA trials. Simultaneously, this subgroup also remains heavily underrepresented in GOA trials compared to the real-world patient population. These findings highlight the need for improvements in both trial design and outcome reporting standards for older adults.

## Simple Summary

Older adults are commonly excluded from drug trials, causing physicians to make treatment decisions based on younger and fitter patients, which may lead to inadequate treatment of older patients. This study assesses publications from practice-changing drug trials in gastroesophageal adenocarcinoma from 2010 to 2024 for the inclusion of older adults and reporting of outcomes for this subgroup. Only 40.1% of patients included in the 13 trials assessed were classified as an older adult. All but one trial excluded patients with a poor functional baseline, as measured by an Eastern Cooperative Oncology Group performance status of ≥2. Apart from the primary aims of the trials, all other outcomes, including secondary aims, quality of life, toxicity and baseline patient characteristics, were poorly reported. No specific measurement tools for older patients were used in any of the trials. This study highlights deficits in both the inclusion and reporting of outcomes for older adults.

## 1. Introduction

The cancer patient population is ageing, with 60% of newly diagnosed cancers in the next decade predicted to occur in patients >65 years of age globally, reaching up to 70% in developed nations [1,2]. The clinical management of older adults with cancer is complicated by the increased likelihood of underlying frailty and multimorbidity [3,4], as well as age-associated differences in priorities for treatment, including a greater emphasis on quality of life and functional outcomes [5,6].

Despite the frequency of cancer diagnoses in older patients and the complexity of giving systemic anti-cancer therapies in this age group, there is a prominent lack of knowledge regarding their treatment outcomes. This is partly due to the underrepresentation of older adults in clinical trials, with recent estimations that only around one third of trial participants are older adults [7,8]. At the same time, there is underreporting of outcomes from those older adults who are enrolled in these trials [7,9].

Older adults are a heterogeneous patient group, and gaps in our knowledge regarding how risk factors such as frailty and co-morbidity interact with cancer treatments lead to both overtreatment and undertreatment of older adults with cancer [10,11]. Indeed, increased age and co-morbidities have been associated with poorer cancer outcomes in terms of both survival and functional outcome [12].

This is particularly relevant to gastric, gastroesophageal junction, and oesophageal (GO) cancers due to the significant proportion of older adults diagnosed [13] and the prevalence of frailty and co-morbidity [14,15]. The focus of this study will be gastroesophageal adenocarcinoma (GOA) as it is the most common subtype in the United Kingdom, responsible for 80% of diagnoses [16]. Disparities in health outcomes between younger and older patients have been observed in GOA [17,18]. Older gastro-oesophageal cancer patients were found to benefit less in terms of overall survival (OS) from neoadjuvant treatment compared to younger patients [17]. In gastric cancer, a widening survival gap between younger and older patients was identified and linked to age-related differences in management with surgery and/or chemotherapy [18].

In the past decade, the treatment landscape for GOA has dramatically changed with the introduction of biomarkers, immune checkpoint inhibitors and targeted therapies. Backbone chemotherapy plus or minus immunotherapy or targeted therapies remains the standard of care for the majority of GOA patients [19,20]. The lack of inclusion of older patients in clinical trials and inadequate subgroup reporting has led to a lack of clinical evidence to support treatment decisions in older patients with regard to these novel cancer therapeutics [7].

This study therefore aims to examine the rate and reliability of reporting of subgroup data regarding older adults recruited to registration clinical trials in gastroesophageal cancers between 2010 and 2024. The availability and completeness of the reporting of protocol-defined endpoints, baseline characteristics, toxicity, and health-related quality of life (HRQOL) domains for older adults were assessed.

## 2. Materials and Methods

This review was performed in accordance with the PRISMA (Preferred Reporting Items for Systematic Reviews and Meta-Analyses) guidelines. The protocol has not been registered, as it is based on a previously published methodology [21]. The PRISMA checklist can be found in the Supplementary Materials.

### 2.1. Trial and Publication Sample

**Figure 1:**
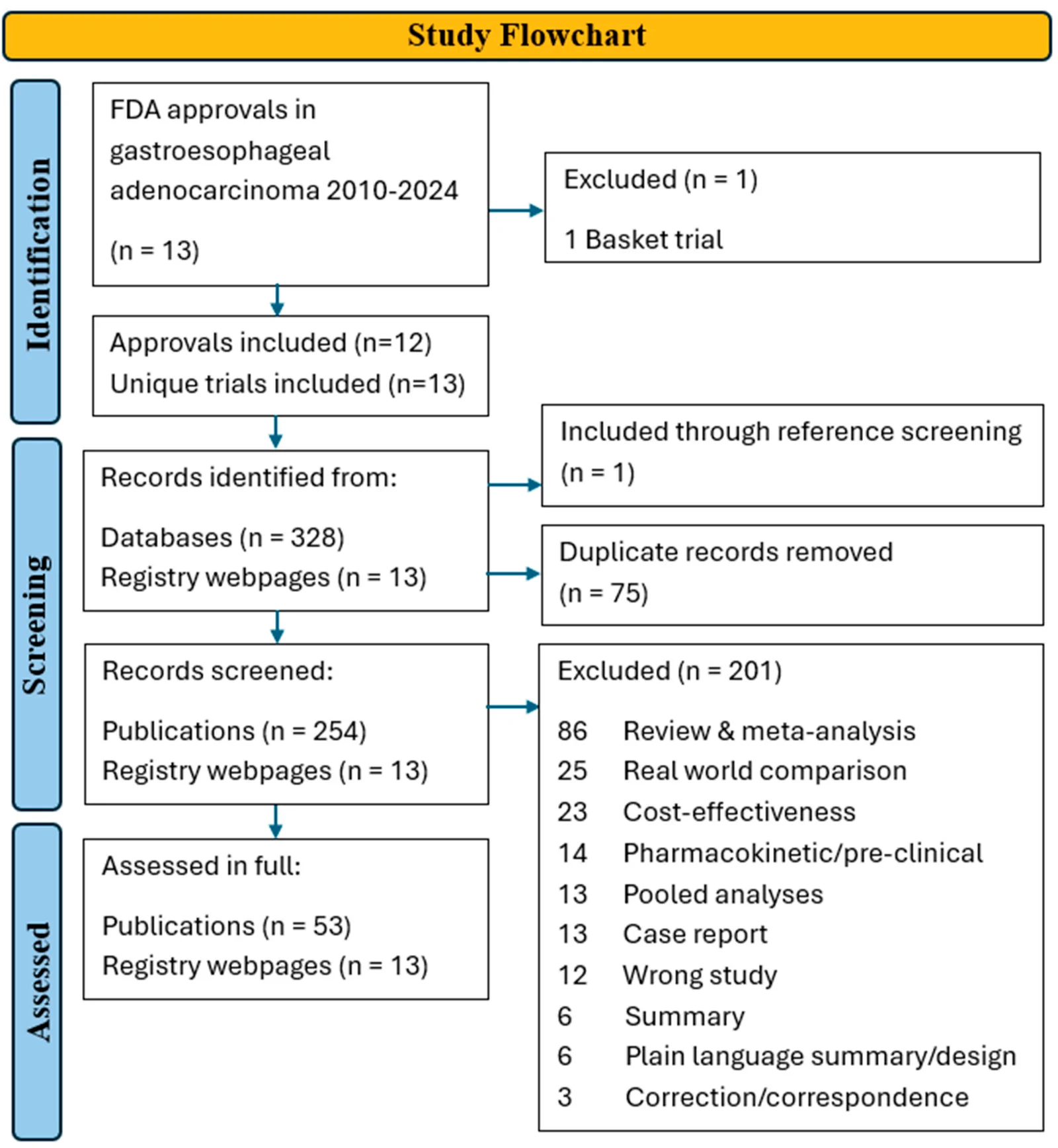
Study flow diagram.

The United States Food and Drug Administration (FDA) oncology approvals and accelerated approvals in gastric, gastro-oesophageal junction and oesophageal adenocarcinoma (GOA) granted between 1 January 2010 and 31 December 2024 were retrieved from publicly available FDA sources [22] (Figure 1). One trial, DESTINY-PanTumor02 [23], was excluded as it was a basket trial which included a limited number of gastroesophageal adenocarcinoma patients, which meant data could not be sufficiently extrapolated. Trials including both adenocarcinomas and squamous cell carcinomas which reported outcomes for the adenocarcinoma subgroup separately, such as CHECKMATE-577 [24] and KEYNOTE-590 [25], were included (Supplementary Table S1). The exploratory cohort of DESTINY-Gastric01 was excluded (*n* = 44) [26], as well as the ipilimumab arm of CHECKMATE-649, as it closed early [27].

Individual trial-level PUBMED searches were conducted on 26 February 2026 for primary and secondary trial-associated publications according to trial registration number (indicative terms: “NCT03504397”[All Fields]) and by both trial and drug name (indicative terms: “Zolbetuximab”[All Fields] AND “SPOTLIGHT”[All Fields]). Data and publication references indexed on the trial’s clinicaltrials.gov repository were also included (Supplementary Table S2). The inclusion of clinicaltrials.gov repositories allowed cross-referencing of both protocol endpoints and published results with the peer-reviewed publications.

Search results were reviewed using Covidence by IT and MK for inclusion and exclusion by title and abstract, followed by full-text review. Inclusion and exclusion criteria are denoted in Table 1. Due to significant heterogeneity in the definition of ‘older patient’, we used ≥65 as the definition if none were applied; however, where available, the trials’ own definition of older subgroup was used. References of included studies were reviewed for further primary or secondary publications. Conflicts were resolved by consensus.

### 2.2. Definition of Efficacy Endpoints

Protocol-defined endpoints were extracted from the most recent study protocol and cross-referenced with the clinicaltrials.gov repository (Supplementary Table S2). Primary and secondary endpoints were included, except those relating to pharmacokinetics or safety. Exploratory endpoints (*n* = 1) were excluded from analysis. Baseline characteristics, toxicity and HRQOL data were examined separately.

Where trials assessed efficacy either across multiple timepoints or centrally versus by local investigators, these were considered a single endpoint. Conversely, where primary or secondary endpoints were defined as assessing pre-determined subpopulations (e.g., overall survival (OS) in the intention-to-treat population versus programmed death-ligand 1 (PDL1) >5 population), these were considered as separate endpoints. Immature efficacy endpoints with no interim data were excluded (*n* = 2).

### 2.3. Data Extraction

For each trial, data regarding trial demographics and outcomes were collected. Additionally, the completeness of the reporting of outcomes for older cancer patients was recorded for the baseline patient characteristics, primary and secondary efficacy outcomes, HRQOL assessments, and toxicity. All trials included in this study applied a definition of older subgroup; all were defined as a minimum age of 65 or ≥65 by all trials except one trial, which applied a threshold of >60 years.

Data extraction, synthesis and presentation were conducted by IT in Excel (Version 2602). Independent extraction of data was performed by MK, with review of the synthesis and presentation of the data. Conflicts were resolved by discussion with a third reviewer, MAB. The level of outcome reporting was analysed with regard to the older subgroups only. Data completeness of primary and secondary efficacy endpoints was defined according to the criteria established by Chan et al. [29] and further developed by MacEchoagain and Battisti [30]. These outcomes were reported as complete, partial, qualitative or quantitative based on availability of sample size, effect size and measures of precision (Table 2). These thresholds were set according to a previously published methodology [21], which increases reproducibility and enables direct comparison of results with previously published outcomes.

Baseline characteristics, toxicity and HRQOL data are multidimensional domains. Therefore, to assess if adequately reported for older adults, these data were assessed using minimum data thresholds (Table 3). This prevented a skewed representation of outcome reporting in cases where a single toxicity domain was reported compared to a full dataset.

## 3. Results

### 3.1. Trial and Study Characteristics

Twelve FDA approvals and accelerated approvals met the inclusion criteria for this study, leading to the inclusion of thirteen unique gastric, gastro-oesophageal junction and oesophageal adenocarcinoma (GOA) trials. From a total of 254 publications, a total of 201 publications were excluded, with reasoning included in Figure 1. Data extraction was completed from 53 publications and 13 clinicaltrial.gov registry pages (Supplementary Tables S2 and S3).

Trial characteristics are shown in Table 4. Most included trials were phase 3 (84.6%) and in the palliative setting (92.3%). Monoclonal antibodies and checkpoint inhibitors were studied in 92.3% of trials.

A total of 9320 individuals were enrolled across the thirteen trials included in this study, with a mean of 717 per trial. Using the study’s own definition of older adult subgroups (e.g., >65 or >60), 40.1% or 3736 patients were considered of old age. Only one trial actively recruited patients with an ECOG status of 2, with two further studies reporting retrospective classification of patients to a PS of 2, representing a total of 0.7% of the total patient population. ECOG status of 0 and 1 represented 42.2% and 57.5% of the total population, respectively. Two trials published unpooled older subgroup findings in a dedicated secondary publication [31,32].

### 3.2. Efficacy Endpoint Reporting

A total of 21 primary endpoints were identified across all studies. Five studies had more than one primary (i.e., co-primary endpoints). Primary endpoints included OS (47.6%), progression-free survival (PFS) (33.3%), objective response rate (ORR) (14.3%) and disease-free survival (4.8%). Nine trials (69.2%) completely reported all primary endpoints among older populations. Overall, 14 primary endpoints (66.7%) were completely reported, with two partial reports (9.5%) and five unreported endpoints (23.8%) (Table 5).

Data were immature for two secondary endpoints and one endpoint related to an exploratory cohort; thus, 65 secondary endpoints were considered in this analysis. Only thirteen secondary endpoints were complete (20.0%) with one partial report (1.5%), one qualitative report (1.5%) and 50 unreported (76.9%). There was no study that fully reported all secondary endpoints for older adults (Supplementary Tables S4 and S5).

### 3.3. Key Efficacy Points

**Figure 2:**
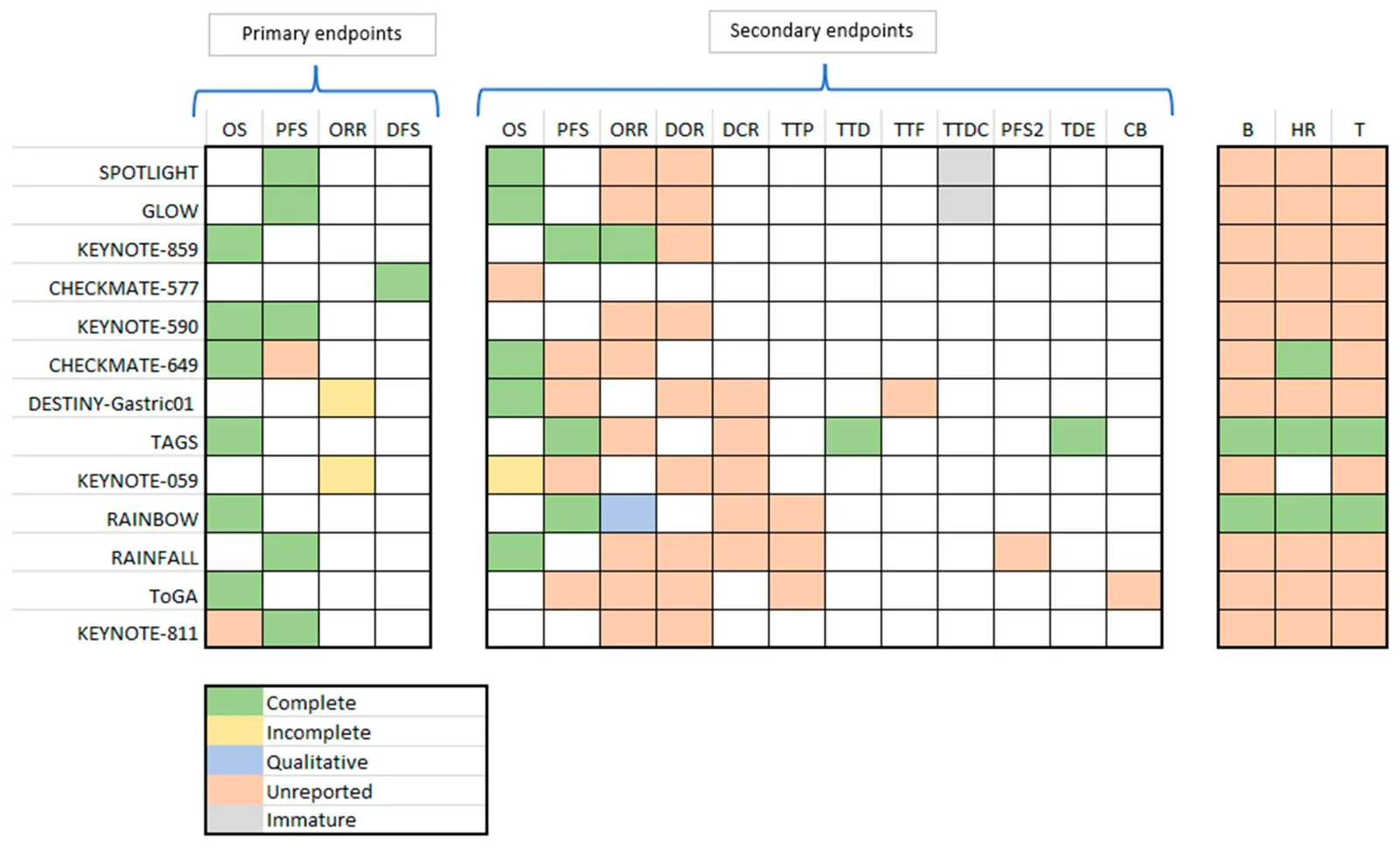
Completeness of efficacy endpoint reporting for older adults according to individual trials. OS—overall survival. PFS—progression-free survival. ORR—objective response rate. DFS—disease-free survival, DOR—duration of response, DCR—disease control rate, TTP—time to progressive disease, TTF—time to treatment failure, TTCD—time to confirmed deterioration, PFS2—progression-free survival measured from randomisation to progression (or death) on second-line systemic therapy, TDE—time to treatment discontinuation, CB—percentage with clinical benefit, B—baseline characteristics, HR—health-related quality of life, T—toxicity.

All thirteen included studies assessed overall survival (OS) as either a primary or secondary endpoint. Of these, ten studies (76.9%) fully reported OS amongst older patients. Two studies did not report any OS data, whilst one study completely reported OS but only for one cohort of the study (Figure 2).

Twelve studies (92.3%) assessed progression-free survival (PFS). A total of four studies (33.0%) did not report any PFS outcomes for older patients; however, all remaining studies reported this data completely (Figure 2).

### 3.4. Baseline Characteristics, Toxicity, and Health-Related Quality of Life

All included studies presented quantitative summaries of participants’ ages using either mean and standard deviation, or median and range, as well as reporting age-stratified enrolment data according to one or more defined age thresholds. One study defined their older adult population as >60 [33], two trials defined it as >65 [34,35], and all other studies as ≥65. Four studies reported several age thresholds, including a subgroup of >75 or ≥75 [31,32,34,35]. Reporting of baseline characteristics of the older adult subgroup was completed in only two studies (15.4%) [31,32]. None of the studies included geriatric-specific baseline assessments.

Twelve studies (92.3%) assessed HRQOL outcomes using validated tools among the full cohort, although none were specific to older adults. Three studies (25.0%) reported HRQOL outcomes for at least one validated tool amongst the older adult population [31,32,36]. All studies reported toxicities among the full study population, but only two studies (15.4%) completely reported toxicity outcomes for older patients [31,32], with the remaining eleven studies (84.6%) not reporting any subgroup data.

## 4. Discussion

This study sought to ascertain the representation and rate of outcome reporting for older adults in practice-changing trials in GOA over the last 14 years. We have identified significant limitations to the representation of older and frailer adults in practice-changing RCTs compared to the real-world cancer patient population. We also found gaps in the reporting of baseline demographic data, secondary efficacy outcomes, toxicity and quality of life metrics amongst the older adults recruited. The reporting of primary efficacy outcomes and, where included either as a primary or secondary efficacy outcome, OS and PFS were generally adequate across the included trials. This mirrors standards of outcome reporting in older adults in trials focusing on different tumour sites [21,30].

Our data highlight the underrepresentation of older cancer patients in RCTs, with only 40.1% of the total trial population consisting of older adults. The mean age of patients included in the GOA trials was 57.6–63.3 years [37,38,39,40,41,42,43,44,45,46,47,48,49]. This is in stark contrast to the real-world patient population, where the mean age at diagnosis is 72 years and 60% of new diagnoses occur in patients aged >70 years [16]. The lack of inclusion of older adults in RCTs is not limited to GOA; in all trials registered with the FDA, only 24% of patients are >70 years of age [8,50,51]. Thus, there is significant age-skewing towards a younger patient population in RCTs. This can be explained through several barriers that older adults experience for their inclusion in RCTs, including trial availability and eligibility, perceived frailty and co-morbidities affecting treatment toxicities, and treatment preferences of the patient [50,51].

The patient population included in these trials is not only younger, but they also have a better functional baseline. Most patients had an ECOG PS of 1 (57.5%), followed by ECOG PS of 0 (42.2%). In comparison, real-world GOA patient characteristics show that 28.7% have an ECOG status of 2, with ECOG status of 1 and 0 occurring in 33.2% and 38.1%, respectively [13]. Of the 13 trials included, only one trial actively recruited patients with an ECOG status of 2, resulting in a total of 63 patients (0.7%) with an ECOG PS 2 across our study. Therefore, the limited number of older adults that are included in these trials are highly selected for a good functional baseline. Drawing conclusions from a patient population with fewer functional impairments and co-morbidities can lead to underrepresentation of treatment toxicities in older cancer patients [52,53].

There is an additional difficulty in estimating the degree of age and functional skewing in these trials due to shortcomings in outcomes reporting. Firstly, there was a marked inconsistency in the age threshold that individual trials apply to define older adults. Ten trials used ≥65 (76.9%), two trials used >65 (15.4%) and a single trial used >60 (7.7%) as their boundary for defining older adults. This introduces further difficulty in the interpretation of the limited data available. Secondly, there was limited reporting of baseline characteristics of older adults. Only two studies reported such baseline characteristics, and whilst noting they were generally similar across younger and older age groups, increased rates of renal impairment, a greater proportion of patients with ECOG status of 1, a greater number of prior therapies [31] and differences in histological subtype [32] were seen. This further illustrates the gap in baseline function between trial and real-world patient populations.

Perceived frailty and co-morbidities affecting treatment tolerance are common reasons for the exclusion of older adults from clinical trials [54,55]. However, chronological age, performance status and physician judgement can be poor indicators of frailty and co-morbidities in older adults, creating a need for more detailed frailty screening and consideration of a comprehensive geriatric assessment [56,57,58,59,60,61]. The feasibility and validity of implementing such geriatric-specific assessments are strongly supported by the literature [56,59,62,63,64,65]. Unfortunately, despite this body of evidence, none of the trials assessed in this study included geriatric-specific baseline assessments.

HRQOL assessment forms an important part of a comprehensive geriatric assessment in its ability to monitor the impact of cancer [66,67]. Unfortunately, HRQOL outcomes for older cancer patients were also poorly reported, with only three studies publishing complete data [31,32,36]. However, these studies use HRQOL instruments that are not validated specifically for older adults and often ignore the complex needs of older adults with cancer [66]. An increased focus on the maintenance of social functioning, physical health, mental health and cognitive changes is required in this patient group [57,68]. Validated geriatric-specific instruments such as the EORTC QLQ-ELD14 questionnaire [69] have been developed for this purpose.

The implementation of geriatric assessments and interventions alongside patient-reported symptomatic toxicity reporting in older patients can reduce the prevalence and severity of toxicities [70,71]. Additionally, there have been significant developments in the field of chemotherapy toxicity prediction tools for older cancer patients [72]. Innovative trial design, including but not limited to implementation of patient-centred assessments, dose adjustments, and outcomes measurements for frail and older patients, such as in the GO2 RCT [28], should be more widely implemented. However, this study shows a lack of implementation of geriatric-specific (baseline) toxicity assessments, as well as reporting of toxicity outcomes, with only two trials publishing toxicity data for older subgroups. The lack of integration of baseline geriatric assessments, HRQOL and toxicity reporting in clinical trials has resulted in a lack of age-specific evidence amongst newer forms of treatment such as immunotherapy [73].

To address both issues of underrepresentation and underreporting of older cancer patients highlighted above, we would propose the following. Barriers currently excluding older patients from clinical trials must be systematically addressed, which can be achieved through relaxing inclusion criteria to include patients with an ECOG status of 2 and by implementing geriatric-specific baseline assessments such as the ASCO Practical Geriatric Assessment [74]. Additionally, we would propose a minimum reporting framework for future oncology registration trials which should report efficacy, toxicity and HRQOL outcomes for planned ≥65 and ≥75 subgroups with sufficient power. As part of this, mandatory baseline and interval geriatric assessments should be reported, geriatric-specific measurements such as time to functional decline or treatment toxicity should be incorporated as efficacy endpoints, and geriatric-specific HRQOL tools should be used, such as the EORTC QLQ-ELD14 for HRQOL outcomes [69]. These improvements in outcome reporting could mitigate the current need for follow-up studies in elderly patients, particularly relevant to treatment tolerance and toxicities, including dose-escalation studies such as GO2 [28]. Regulatory agencies such as the FDA can play a crucial role in accelerating improvements in reporting standards through implementing the above as protocol requirements for RTCs.

To our knowledge, this is the first study to assess the representation and outcome reporting for older GOA patients in practice-changing RCTs. Our study is strengthened by the fact that it not only captures all relevant primary and secondary publications but also uses the grey literature by including the clinicaltrial.gov repository. Furthermore, the trials included capture the changing wider landscape in oncology with approvals mainly concerning monoclonal antibodies and checkpoint inhibitors, and several studies implementing planned analysis of subgroups based on PDL1 status.

However, there are several limitations to the methodology adopted here. Trials included in the study were generated from FDA (accelerated) approvals only, which may have omitted trials that have been considered as ‘practice-changing’ by other regulatory bodies. Secondly, our database search was restricted to PubMed and clinicaltrials.gov to capture primary and secondary publications without introducing too much noise from real-world studies, prospective studies and unpublished results, which were not considered in this study. Additionally, not enough time may have elapsed between the conclusion or initial publication of the most recent trials for planned post hoc analyses to be published yet. Therefore, the publications included here may not be fully representative of all planned outcome reporting in relation to the included trials. Lastly, two trials included in this study recruited patients with both adenocarcinoma and squamous cell carcinoma (SSC) [24,25]. A total of 778 patients, or 8.3% of the total patient population across all trials, had an SSC, and whilst these trials report outcomes for the overall population separately for this subgroup, this was not the case for the reporting of outcomes for older adults. No separate sensitivity analysis could be performed to mitigate this limitation as we did not have access to the raw data.

## 5. Conclusions

In summary, our study shows that older and frailer patients are insufficiently represented in GOA RCTs compared to the real world. It furthermore highlights deficiencies in both the standards of outcome reporting for older subgroups and in the use of geriatric-specific assessments and measurement tools. However, there is a growing awareness of these issues, as evidenced by the publication of a formal strategy for the inclusion of older cancer patients by ESMO/SIOG [75]. Further change is required to support clinicians in making treatment decisions for older adults in the real world, particularly relating to checkpoint inhibitors and monoclonal antibodies evaluated in the trials included in this study. A growing body of literature concerning this issue has led to the development of both clinical guidelines specific to older cancer patients [76,77] and recommendations for inclusive RCT design [50,78,79]. Whilst there is growing awareness and evidence of initial changes in standard practice regarding the inclusion of older patients in clinical trials, there remains a strong need for the standardisation of geriatric-specific outcome reporting to support treatment decisions in this subgroup, particularly for novel anti-cancer therapeutics.

## Figures and Tables

**Table 1:** Inclusion and exclusion criteria for publications assessed.

Domain	Inclusion	Exclusion
Study characteristics	• Primary study reports • Secondary publications • Clinicaltrial.gov database	• Plain language summaries & study design • Review papers & meta- analyses • Pooled analyses without new primary data • Conference reviews & corrections & correspondence • Case reports • Summary
Population	• Gastric, gastro-oesophageal junction and oesophageal adenocarcinoma	• Oesophageal squamous cell carcinoma • Trials specifically in older adults
Intervention/ exposure	• Clinical trials leading to a US Food and Drug Administration (FDA) full approval or accelerated approval between 2010 and 2024	• Dose de-escalation studies (i.e., GO2 [28])
Outcomes	• Primary and secondary efficacy outcomes • Baseline characteristics • Toxicity • Health-related quality of life	• Cost-effectiveness analysis • Pharmacokinetics/pre-clinical focus • Real-world comparison

**Table 2:** Efficacy endpoint assessment. Adapted from [29,30].

Level of Reporting	Reported Data	Sufficient for Inclusion in Meta-Analysis
Complete	(1) Number of participants per group (2) Effect size (3) Precision or precise *p*-value for continuous data	Yes
Partial	Effect size or precision (±*p*-value, ±sample size)	No
Qualitative	*p*-value ± sample size	No
Unreported	Not available	No

**Table 3:** Baseline characteristics, toxicity, and HRQOL endpoints. Adapted from [29,30].

Domain	Minimum Threshold
Baseline characteristics	Performance status OR co-morbidities AND ≥1 Key prognostic OR predictive factor(s) *
Toxicity	≥3 Key toxicity domain(s) by organ site OR Overall G3 toxicity AND ≥1 key toxicity domain *
HRQOL	≥1 validated HRQOL instrument(s)
**Level of Reporting**	**Reported Data**
Complete	Meets minimum threshold, including numerical data sufficient for inclusion in meta-analysis
Partial	Meets minimum threshold, including numerical data insufficient for inclusion in meta-analysis
Qualitative	Meets minimum threshold, without numerical data
Unreported	Does not meet minimum threshold

* Key prognostic OR predictive factors: stage, grade, genetic factors (e.g., HER2). Key toxicity domains (by organ site): haematological, gastrointestinal, dermatological, etc.

**Table 4:** Characteristics of included studies.

	Number	Percentage
**Phase**		
II	2	15.4%
III	11	84.6%
**Disease site**		
GC and GEJC	10	76.9%
EAC and GEJC	2	15.3%
GC, EAC and GEJC	1	7.7%
**Histology**		
Adenocarcinoma	11	84.6%
Adenocarcinoma & SSC	2	15.4%
**Setting**		
Advanced/metastatic	12	92.3%
Adjuvant	1	7.7%
**Molecular characteristics**		
HER2-positive	3	23.1%
HER2-negative	5	38.5%
CDLN18.2 positive	2	15.4%
**Drug type**		
Monoclonal antibody *	6	46.2%
Checkpoint inhibitor *	5	38.5%
Monoclonal antibody & checkpoint inhibitor *	1	7.7%
Chemotherapy	1	7.7%
**Enrolment**		
N (mean, 95% CI)	716.9	462.7–971.2
100–250	1	7.7%
250–500	1	7.7%
500–750	8	61.5%
750–1000	1	7.7%
>1000	2	15.4%
**Population**		
Total	9320	100%
Older adult ^	3736	40.1%
ECOG 0 ^±^	3690	42.2%
ECOG 1 ^±^	5025	57.5%
ECOG 2	63	0.7%

* In combination with chemotherapy. ^ Definition of ≥65 used by all trials with the exception of SPOTLIGHT and GLOW (>65) and ToGA (>60). ^±^ ToGA trial excluded, which reported 90.2% of patients as ECOG 0/1. GC = gastric cancer. EAC = oesophageal cancer. GEJ = gastro-oesophageal junction cancer. SSC = squamous cell carcinoma. ECOG = Eastern Cooperative Oncology Group performance status.

**Table 5:** Completeness of reporting for older subgroup by category across all included studies.

	N	Complete (%)	Partial (%)	Qualitative (%)	Unreported (%)
Primary endpoints	21	14 (66.7%)	2 (9.5%)	0 (0.0%)	5 (23.8%)
Secondary endpoints	65	13 (20.0%)	1 (1.5%)	1 (1.5%)	50 (76.9%)
Baseline	13	2 (15.4%)	0 (0.0%)	0 (0.0%)	11 (84.6%)
Toxicity	13	2 (15.4%)	0 (0.0%)	0 (0.0%)	11 (84.6%)
HRQOL *	12	3 (25.0%)	0 (0.0%)	0 (0.0%)	9 (75.0%)

* HRQOL = health-related quality of life.

## Data Availability

The original contributions presented in this study are included in the article/Supplementary Materials. Further inquiries can be directed to the corresponding author.

## Supplementary Materials

The following supporting information can be downloaded at: https://www.mdpi.com/article/10.3390/cancers18172747/s1, Table S1: Overview of trials included in study; Table S2: Trial repositories and protocols utilised; Table S3: Included publications; Table S4: Overview of trial outcomes; Table S5: Completeness of outcome reporting; PRISMA 2020 Checklist.

## Abbreviations

The following abbreviations are used in this manuscript:

CB	Clinical benefit
DFS	Disease-free survival
DCR	Disease control rate
DOR	Duration of response
EAC	Oesophageal cancer
ECOG	Eastern Cooperative Oncology Group
FDA	Food and Drug Administration
GC	Gastric cancer
GEJ	Gastro-oesophageal junction cancer
GOA	Gastric, gastro-oesophageal junction and oesophageal adenocarcinoma
HRQOL	Health-related quality of life
OS	Overall survival
ORR	Objective response rate
PDL1	Programmed death-ligand 1
PFS	Progression-free survival
PFS2	Progression-free survival measured from randomisation to progression (or death) on second-line systemic therapy
PS	Performance status
RCT	Randomised controlled trials
SSC	Squamous cell carcinoma
TDE	Time to treatment discontinuation
TTCD	Time to confirmed deterioration
TTF	Time to treatment failure
TTP	Time to progressive disease

## References

[1] Pilleron S., Sarfati D., Janssen-Heijnen M., Vignat J., Ferlay J., Bray F., Soerjomataram I. (2018). Global cancer incidence in older adults, 2012 and 2035: A population-based study. Int. J. Cancer.

[2] Garner W.B., Smith B.D., Ludmir E.B., Wakefield D.V., Shabason J., Williams G.R., Martin M.Y., Wang Y., Ballo M.T., VanderWalde N.A. (2022). Predicting future cancer incidence by age, race, ethnicity, and sex. J. Geriatr. Oncol..

[3] Ethun C.G., Bilen M.A., Jani A.B., Maithel S.K., Ogan K., Master V.A. (2017). Frailty and cancer: Implications for oncology surgery, medical oncology, and radiation oncology. CA Cancer J. Clin..

[4] Chen W., Altshuler R.D., Daschner P., Morales C.S., St Germain D.C., Guida J., Prasanna P.G.S., Buchsbaum J.C. (2024). Older adults with cancer and common comorbidities—Challenges and opportunities in improving their cancer treatment outcomes. J. Natl. Cancer Inst..

[5] Soto-Perez-De-Celis E., Dale W., Katheria V., Kim H., Fakih M., Chung V.M., Lim D., Mortimer J., Chien L.C., Charles K. (2024). Outcome prioritization and preferences among older adults with cancer starting chemotherapy in a randomized clinical trial. Cancer.

[6] Grellety T., Bellera C., Cantarel C., Mertens C., Cabart M., Roubaud G., Chantecaille M.-C., Desclos H., Souyris C., Bouchaert P. (2025). Important aspects of care and priorities of older patients with cancer: The PRIORITY multicenter cohort study. J. Geriatr. Oncol..

[7] Chow R., Lage D.E., Williams G.R., Sedrak M.S., Greer J.A., Temel J.S., Nipp R.D. (2022). Representation and Outcomes of Older Adults in Practice-Changing Oncology Trials in the Era of Novel Therapies: A Guideline Appraisal. J. Natl. Compr. Cancer Netw..

[8] Singh H., Kanapuru B., Smith C., Fashoyin-Aje L.A., Myers A., Kim G., Pazdur R. (2017). FDA analysis of enrollment of older adults in clinical trials for cancer drug registration: A 10-year experience by the U.S. Food and Drug Administration. J. Clin. Oncol..

[9] Brintzenhofeszoc K., Krok-Schoen J.L., Canin B., Parker I., MacKenzie A.R., Koll T., Vankina R., Hsu C.D., Jang B., Pan K. (2020). The underreporting of phase III chemo-therapeutic clinical trial data of older patients with cancer: A systematic review. J. Geriatr. Oncol..

[10] DuMontier C., Loh K.P., Soto-Perez-De-Celis E., Dale W. (2021). Decision Making in Older Adults with Cancer. J. Clin. Oncol..

[11] DuMontier C., Loh K.P., Bain P.A., Silliman R.A., Hshieh T., Abel G.A., Djulbegovic B., Driver J.A., Dale W. (2020). Defining Undertreatment and Overtreatment in Older Adults with Cancer: A Scoping Literature Review. J. Clin. Oncol..

[12] Chen R.C., Royce T.J., Extermann M., Reeve B.B. (2012). Impact of Age and Comorbidity on Treatment and Outcomes in Elderly Cancer Patients. Semin. Radiat. Oncol..

[13] Baxter M.A., Khan K.S., Gall L.S., Samuelson C., McCollum C., Chuntamongkol R., Narramneni L.R., Al-Zuabi M., Bryce G., Shareef H.E.J. (2023). Diagnosis, treatment, and outcome of patients with oesophagogastric cancer during the COVID-19 pandemic: National study. Br. J. Surg..

[14] Handforth C., Clegg A., Young C., Simpkins S., Seymour M.T., Selby P.J., Young J. (2014). The prevalence and outcomes of frailty in older cancer patients: A systematic review. Ann. Oncol..

[15] Chang W.H., Lai A.G. (2024). Pan-cancer analyses of the associations between 109 pre-existing conditions and cancer treatment patterns across 19 adult cancers. Sci. Rep..

[16] Pucher P.H., Park M.H., Cromwell D.A., Crosby T.C., Thomas B., Trudgill N., Wahedally M., Maynard N., Gossage J.A. (2023). Diagnosis and treatment for gastro-oesophageal cancer in England and Wales: Analysis of the National Oesophago-Gastric Cancer Audit (NOGCA) database 2012–2020. Br. J. Surg..

[17] Rompen I.F., Crnovrsanin N., Nienhüser H., Neuschütz K., Fourie L., Sisic L., Müller-Stich B.P., Billeter A.T. (2023). Age-dependent benefit of neoadjuvant treatment in adenocarcinoma of the esophagus and gastroesophageal junction: A multicenter retrospective observational study of young versus old patients. Int. J. Surg..

[18] Nelen S.D., Verhoeven R.H.A., Lemmens V.E.P.P., de Wilt J.H.W., Bosscha K. (2017). Increasing survival gap between young and elderly gastric cancer patients. Gastric Cancer.

[19] Sexton R.E., Al Hallak M.N., Diab M., Azmi A.S. (2020). Gastric cancer: A comprehensive review of current and future treatment strategies. Cancer Metastasis Rev..

[20] Kunene V., Ding M., Yap M., Griffiths E.A., Taniere P., Fackrell D., Butler S., Contino G. (2024). Prognostic markers in oesophageal and gastric cancer review. Are they ready for clinical practice?. ESMO Gastrointest. Oncol..

[21] Mac Eochagain C., Power R., Sam C., Gonzalez-Senac N.M., Walsh D., Roy M., Battisti N.M.L. (2024). Inclusion, characteristics, and reporting of older adults in FDA registration studies of immunotherapy, 2018–2022. J. Immunother. Cancer.

[22] U.S. Food and Drug Administration Oncology (Cancer)/Hematologic Malignancies Approval Notifications. https://www.fda.gov/drugs/resources-information-approved-drugs/oncology-cancerhematologic-malignancies-approval-notifications.

[23] Meric-Bernstam F., Makker V., Oaknin A., Oh D.-Y., Banerjee S., González-Martín A., Jung K.H., Ługowska I., Manso L., Manzano A. (2024). Efficacy and Safety of Trastuzumab Deruxtecan in Patients with HER2-Expressing Solid Tumors: Primary Results from the DESTINY-PanTumor02 Phase II Trial. J. Clin. Oncol..

[24] Kelly R.J., Ajani J.A., Kuzdzal J., Zander T., Van Cutsem E., Piessen G., Mendez G., Feliciano J., Motoyama S., Lievre A. (2021). Adjuvant Nivolumab in Resected Esophageal or Gastroesophageal Junction Cancer. N. Engl. J. Med..

[25] Sun J.-M., Shen L., Shah M.A., Enzinger P., Adenis A., Doi T., Kojima T., Metges J.-P., Li Z., Kim S.-B. (2021). Pembrolizumab plus chemotherapy versus chemotherapy alone for first-line treatment of advanced oesophageal cancer (KEYNOTE-590): A randomised, placebo-controlled, phase 3 study. Lancet.

[26] Shitara K., Bang Y.-J., Iwasa S., Sugimoto N., Ryu M.-H., Sakai D., Chung H.-C., Kawakami H., Yabusaki H., Lee J. (2020). Trastuzumab Deruxtecan in Previously Treated HER2-Positive Gastric Cancer. N. Engl. J. Med..

[27] Shitara K., Ajani J.A., Moehler M., Garrido M., Gallardo C., Shen L., Yamaguchi K., Wyrwicz L., Skoczylas T., Bragagnoli A.C. (2022). Nivolumab plus chemotherapy or ipilimumab in gastro-oesophageal cancer. Nature.

[28] Hall P.S., Swinson D., Cairns D.A., Waters J.S., Petty R., Allmark C., Ruddock S., Falk S., Wadsley J., Roy R. (2021). Efficacy of Reduced-Intensity Chemotherapy with Oxaliplatin and Capecitabine on Quality of Life and Cancer Control Among Older and Frail Patients with Advanced Gastroesophageal Cancer: The GO2 Phase 3 Randomized Clinical Trial. JAMA Oncol..

[29] Chan A.-W., Hróbjartsson A., Haahr M.T., Gøtzsche P.C., Altman D.G. (2004). Empirical evidence for selective reporting of outcomes in randomized trials: Comparison of protocols to published articles. JAMA.

[30] Eochagain C.M., Battisti N.M.L. (2023). Reporting of older subgroups in registration breast cancer trials 2012–2021. Breast Cancer Res. Treat..

[31] Shitara K., Doi T., Hosaka H., Thuss-Patience P., Santoro A., Longo F., Ozyilkan O., Cicin I., Park D., Zaanan A. (2022). Efficacy and safety of trifluridine/tipiracil in older and younger patients with metastatic gastric or gastroesophageal junction cancer: Subgroup analysis of a randomized phase 3 study (TAGS). Gastric Cancer.

[32] Muro K., Cho J.Y., Bodoky G., Goswami C., Chao Y., dos Santos L.V., Shimada Y., Topuzov E., Van Cutsem E., Tabernero J. (2017). Age does not influence efficacy of ramucirumab in advanced gastric cancer: Subgroup analyses of REGARD and RAINBOW. J. Gastroenterol. Hepatol..

[33] Bang Y.-J., Van Cutsem E., Feyereislova A., Chung H.C., Shen L., Sawaki A., Lordick F., Ohtsu A., Omuro Y., Satoh T. (2010). Trastuzumab in combination with chemotherapy versus chemotherapy alone for treatment of HER2-positive advanced gastric or gastro-oesophageal junction cancer (ToGA): A phase 3, open-label, randomised controlled trial. Lancet.

[34] Shitara K., Lordick F., Bang Y.-J., Enzinger P., Ilson D., Shah M.A., Van Cutsem E., Xu R.-H., Aprile G., Xu J. (2023). Zolbetuximab plus mFOLFOX6 in patients with CLDN18.2-positive, HER2-negative, untreated, locally advanced unresectable or metastatic gastric or gastro-oesophageal junction adenocarcinoma (SPOTLIGHT): A multicentre, randomised, double-blind, phase 3 trial. Lancet.

[35] Shah M.A., Shitara K., Ajani J.A., Bang Y.-J., Enzinger P., Ilson D., Lordick F., Van Cutsem E., Plazas J.G., Huang J. (2023). Zolbetuximab plus CAPOX in CLDN18.2-positive gastric or gastroesophageal junction adenocarcinoma: The randomized, phase 3 GLOW trial. Nat. Med..

[36] Lin D., Quan W., Garretson M., Chirikov V., Chen C., Singh P., Davis C., Sugarman R. (2025). Q-TWiST analysis of first-line nivolumab plus chemotherapy versus chemotherapy in patients with advanced gastric cancer, gastroesophageal junction cancer, or esophageal adenocarcinoma from CheckMate 649: 4-year follow-up results. Gastric Cancer.

[37] A Study to Compare Zolbetuximab (IMAB362) and Chemotherapy with Placebo and Chemotherapy in Adults with Gastric Cancer (Spotlight). ClinicalTrials.gov Identifier: NCT03504397. Updated 29 October 2025. NCT03504397.

[38] A Study of Zolbetuximab (IMAB362) Plus CAPOX Compared with Placebo Plus CAPOX as First-Line Treatment of Subjects with Claudin (CLDN) 18.2-Positive, HER2-Negative, Locally Advanced Unresectable or Metastatic Gastric or Gastroesophageal Junction (GEJ) Adenocarcinoma (GLOW). ClinicalTrials.gov Identifier: NCT03653507. Updated 24 October 2025. NCT03653507.

[39] Pembrolizumab (MK-3475) Plus Chemotherapy Versus Placebo Plus Chemotherapy in Participants Gastric or Gastroesophageal Junction (GEJ) Adenocarcinoma (MK-3475-859/KEYNOTE-859). ClinicalTrials.gov Identifier: NCT03675737. Updated 8 July 2025. NCT03675737.

[40] An Investigational Immuno-Therapy Study of Nivolumab or Placebo in Participants with Resected Esophageal or Gastroesophageal Junction Cancer (CheckMate 577). ClinicalTrials.gov Identifier: NCT02743494. Updated 18 November 2025. NCT02743494.

[41] First-line Esophageal Carcinoma Study with Pembrolizumab Plus Chemo vs. Chemo (MK-3475-590/KEYNOTE-590). ClinicalTrials.gov Identifier: NCT03189719. Updated 15 October 2024. NCT03189719.

[42] Efficacy Study of Nivolumab Plus Ipilimumab or Nivolumab Plus Chemotherapy Against Chemotherapy in Stomach Cancer or Stomach/Esophagus Junction Cancer (CheckMate649). ClinicalTrials.gov Identifier: NCT02872116. Updated 7 August 2025. NCT02872116.

[43] DS-8201a in Human Epidermal Growth Factor Receptor 2 (HER2)-Expressing Gastric Cancer [DESTINY-Gastric01]. ClinicalTrials.gov Identifier: NCT03329690. Updated 18 March 2022. NCT03329690.

[44] Study of TAS-102 or Placebo Plus BSC in Patients with Metastatic Gastric Cancer. ClinicalTrials.gov Identifier: NCT02500043. Updated 3 September 2024. NCT02500043.

[45] A Study of Pembrolizumab (MK-3475) in Participants with Recurrent or Metastatic Gastric or Gastroesophageal Junction Adenocarcinoma (MK-3475-059/KEYNOTE-059). ClinicalTrials.gov Identifier: NCT0233541. Updated 8 August 2022. https://clinicaltrials.gov/study/NCT02335411.

[46] A Study of Paclitaxel with or Without Ramucirumab (IMC-1211B) in Metastatic Gastric Adenocarcinoma (RAINBOW). ClinicalTrials.gov Identifier: NCT01170663. Updated 18 September 2019. NCT01170663.

[47] A Study of Ramucirumab (LY3009806) in Combination with Capecitabine and Cisplatin in Participants with Stomach Cancer (RAINFALL). ClinicalTrials.gov Identifier: NCT02314117. Updated 26 August 2021. NCT02314117.

[48] ToGA Study—A Study of Herceptin (Trastuzumab) in Combination with Chemotherapy Compared with Chemotherapy Alone in Patients with HER2-Positive Advanced Gastric Cancer. ClinicalTrials.gov Identifier: NCT01041404. Updated 5 November 2014. NCT01041404.

[49] Pembrolizumab/Placebo Plus Trastuzumab Plus Chemotherapy in Human Epidermal Growth Factor Receptor 2 Positive (HER2+) Advanced Gastric or Gastroesophageal Junction (GEJ) Adenocarcinoma (MK-3475-811/KEYNOTE-811). ClinicalTrials.gov Identifier: NCT03615326. Updated 11 December 2025. NCT03615326.

[50] Sedrak M.S., Freedman R.A., Cohen H.J., Muss H.B., Jatoi A., Klepin H.D., Wildes T.M., Le-Rademacher J.G., Kimmick G.G., Tew W.P. (2020). Older adult participation in cancer clinical trials: A systematic review of barriers and interventions. CA Cancer J. Clin..

[51] Scher K.S., Hurria A. (2012). Under-Representation of Older Adults in Cancer Registration Trials: Known Problem, Little Progress. J. Clin. Oncol..

[52] Lewis J.H., Kilgore M.L., Goldman D.P., Trimble E.L., Kaplan R., Montello M.J., Housman M.G., Escarce J.J. (2003). Participation of Patients 65 Years of Age or Older in Cancer Clinical Trials. J. Clin. Oncol..

[53] Ludmir E.B., Mainwaring W., Lin T.A., Miller A.B., Jethanandani A., Espinoza A.F., Mandel J.J., Lin S.H., Smith B.D., Smith G.L. (2019). Factors Associated with Age Disparities Among Cancer Clinical Trial Participants. JAMA Oncol..

[54] Kornblith A.B., Kemeny M., Peterson B.L., Wheeler J., Crawford J., Bartlett N., Fleming G., Graziano S., Muss H., Cohen H.J. (2002). Survey of oncologists’ perceptions of barriers to accrual of older patients with breast carcinoma to clinical trials. Cancer.

[55] Hurria A., Wong F.L., Villaluna D., Bhatia S., Chung C.T., Mortimer J., Hurvitz S., Naeim A. (2008). Role of Age and Health in Treatment Recommendations for Older Adults with Breast Cancer: The Perspective of Oncologists and Primary Care Providers. J. Clin. Oncol..

[56] Hurria A., Mohile S., Gajra A., Klepin H., Muss H., Chapman A., Feng T., Smith D., Sun C.-L., De Glas N. (2016). Validation of a Prediction Tool for Chemotherapy Toxicity in Older Adults with Cancer. J. Clin. Oncol..

[57] Dale W., Mohile S.G., Eldadah B.A., Trimble E.L., Schilsky R.L., Cohen H.J., Muss H.B., Schmader K.E., Ferrell B., Extermann M. (2012). Biological, Clinical, and Psychosocial Correlates at the Interface of Cancer and Aging Research. J. Natl. Cancer Inst..

[58] Brunello A., Sandri R., Extermann M. (2009). Multidimensional geriatric evaluation for older cancer patients as a clinical and research tool. Cancer Treat. Rev..

[59] Soto-Perez-De-Celis E., Li D., Yuan Y., Lau Y.M., Hurria A. (2018). Functional versus chronological age: Geriatric assessments to guide decision making in older patients with cancer. Lancet Oncol..

[60] Lowsky D.J., Olshansky S.J., Bhattacharya J., Goldman D.P. (2013). Heterogeneity in Healthy Aging. J. Gerontol. Ser. A.

[61] van Walree I.C., Scheepers E., van Huis-Tanja L., Emmelot-Vonk M.H., Bellera C., Soubeyran P., Hamaker M.E. (2019). A systematic review on the association of the G8 with geriatric assessment, prognosis and course of treatment in older patients with cancer. J. Geriatr. Oncol..

[62] Extermann M., Boler I., Reich R.R., Lyman G.H., Brown R.H., DeFelice J., Levine R.M., Lubiner E.T., Reyes P., Schreiber F.J. (2011). Predicting the risk of chemotherapy toxicity in older patients: The Chemotherapy Risk Assessment Scale for High-Age Patients (CRASH) score. Cancer.

[63] Hurria A., Gupta S., Zauderer M., Zuckerman E.L., Cohen H.J., Muss H., Rodin M., Panageas K.S., Holland J.C., Saltz L. (2005). Developing a cancer-specific geriatric assessment: A feasibility study. Cancer.

[64] Hurria A., Togawa K., Mohile S.G., Owusu C., Klepin H.D., Gross C.P., Lichtman S.M., Gajra A., Bhatia S., Katheria V. (2011). Predicting Chemotherapy Toxicity in Older Adults with Cancer: A Prospective Multicenter Study. J. Clin. Oncol..

[65] Hurria A., Akiba C., Kim J., Mitani D., Loscalzo M., Katheria V., Koczywas M., Pal S., Chung V., Forman S. (2016). Reliability, Validity, and Feasibility of a Computer-Based Geriatric Assessment for Older Adults with Cancer. J. Oncol. Pr..

[66] Fitzsimmons D., Gilbert J., Howse F., Young T., Arrarras J.-I., Brédart A., Hawker S., George S., Aapro M., Johnson C.D. (2009). A systematic review of the use and validation of health-related quality of life instruments in older cancer patients. Eur. J. Cancer.

[67] Fitch M.I., Strohschein F.J., Nyrop K. (2021). Measuring quality of life in older people with cancer. Curr. Opin. Support. Palliat. Care.

[68] Seghers P.A.L., Kregting J.A., van Huis-Tanja L.H., Soubeyran P., O’Hanlon S., Rostoft S., Hamaker M.E., Portielje J.E.A. (2022). What Defines Quality of Life for Older Patients Diagnosed with Cancer? A Qualitative Study. Cancers.

[69] Wheelwright S., Darlington A.-S., Fitzsimmons D., Fayers P., Arraras J.I., Bonnetain F., Brain E., Bredart A., Chie W.-C., Giesinger J. (2013). International validation of the EORTC QLQ-ELD14 questionnaire for assessment of health-related quality of life elderly patients with cancer. Br. J. Cancer.

[70] Culakova E., Mohile S.G., Peppone L., Ramsdale E., Mohamed M., Xu H., Wells M., Tylock R., Java J., Loh K.P. (2023). Effects of a Geriatric Assessment Intervention on Patient-Reported Symptomatic Toxicity in Older Adults with Advanced Cancer. J. Clin. Oncol..

[71] Li D., Sun C.-L., Kim H., Soto-Perez-de-Celis E., Chung V., Koczywas M., Fakih M., Chao J., Cabrera Chien L., Charles K. (2021). Geriatric Assessment-Driven Intervention (GAIN) on Chemotherapy-Related Toxic Effects in Older Adults with Cancer: A Randomized Clinical Trial. JAMA Oncol..

[72] Battisti N.M.L., Arora S.P. (2022). An overview of chemotherapy toxicity prediction tools in older adults with cancer: A young international society of geriatric oncology and nursing and allied health initiative. J. Geriatr. Oncol..

[73] Welaya K., Loh K.P., Messing S., Szuba E., Magnuson A., Mohile S.G., Maggiore R.J. (2020). Geriatric assessment and treatment outcomes in older adults with cancer receiving immune checkpoint inhibitors. J. Geriatr. Oncol..

[74] Dale W., Klepin H.D., Williams G.R., Alibhai S.M.H., Bergerot C., Brintzenhofeszoc K., Hopkins J.O., Jhawer M.P., Katheria V., Loh K.P. (2023). Practical Assessment and Management of Vulnerabilities in Older Patients Receiving Systemic Cancer Therapy: ASCO Guideline Update. J. Clin. Oncol..

[75] Baldini C., Mislang A.R.A., Cheung K.-L., Pilleron S., Wildiers H., Rostoft S., Neuendorff N.R., Frelaut M., Kanesvaran R., Papamichael D. (2026). The ESMO/SIOG Cancer in the Elderly Working Group pragmatic strategies for clinical trial designs and endpoints in older adults with cancer. ESMO Open.

[76] Hurria A., Levit L.A., Dale W., Mohile S.G., Muss H.B., Fehrenbacher L., Magnuson A., Lichtman S.M., Bruinooge S.S., Soto-Perez-De-Celis E. (2015). Improving the Evidence Base for Treating Older Adults with Cancer: American Society of Clinical Oncology Statement. J. Clin. Oncol..

[77] Mohile S.G., Dale W., Somerfield M.R., Schonberg M.A., Boyd C.M., Burhenn P.S., Canin B., Cohen H.J., Holmes H.M., Hopkins J.O. (2018). Practical Assessment and Management of Vulnerabilities in Older Patients Receiving Chemotherapy: ASCO Guideline for Geriatric Oncology. J. Clin. Oncol..

[78] Le-Rademacher J., Mohile S., Unger J., Hudson M.F., Foster J., Lichtman S., Perlmutter J., Dotan E., Extermann M., Dodd K. (2022). Trial Design Considerations to Increase Older Adult Accrual to National Cancer Institute Clinical Trials. JNCI Monogr..

[79] Hurria A., Dale W., Mooney M., Rowland J.H., Ballman K.V., Cohen H.J., Muss H.B., Schilsky R.L., Ferrell B., Extermann M. (2014). Designing Therapeutic Clinical Trials for Older and Frail Adults with Cancer: U13 Conference Recommendations. J. Clin. Oncol..

